# Mortality in traffic accidents with older adults in Colombia

**DOI:** 10.1590/S1518-8787.2017051006405

**Published:** 2017-03-07

**Authors:** Angela Maria Segura Cardona, Doris Cardona Arango, Dedsy Yajaira Berbesí Fernández, Alejandra Agudelo Martínez

**Affiliations:** I Grupo de investigación en Epidemiología y Bioestadística. Facultad de Medicina. Universidad CES. Medellín, Colombia

**Keywords:** Aged, Accidents, Traffic, mortality, Potential Years of Life Lost

## Abstract

**OBJECTIVE:**

To analyze the traffic accident mortality in the Colombian older adults during the 1998-2012 period and show the loss of productive years and mortality from this cause.

**METHODS:**

Quantitative study of the trend analysis of deaths in Colombia in traffic accidents, from 1998 to 2012, according to death records and population projected by the Colombian National Administrative Department of Statistics. Frequency distribution profile of the deceased, death rates per hundred thousand inhabitants, potential years of life lost and calculation of excess mortality by age in the over 60 were made.

**RESULTS:**

In the study period 100,758 deaths occurred in traffic accidents, 6,717 annual average, of which 18.5% occurred in people aged 60 years and over. The predominated deaths were men; the risk of dying was 32.15 per hundred thousand people in this age range, with double risk of dying those under 60 years.

**CONCLUSIONS:**

The young population has a higher proportion of deaths, but those over 60 years are at increased risk of death, leading to the need to turn our gaze to the improvement of road infrastructure and standards, to educate the population in self-care and compliance with safety measures and prepare society for an ever more adult population, more numerous and more prone to take risks.

## RESUMEN

**OBJETIVO:**

Analizar la mortalidad por accidente de tránsito en el adulto mayor de Colombia.

**MÉTODOS:**

Estudio cuantitativo del análisis de tendencias de las muertes ocurridas en Colombia por accidentes de tránsito entre 1998 y 2012, según los registros de defunción y la población proyectada para estos años, del Departamento Administrativo Nacional de Estadísticas. Se realizaron distribuciones de frecuencias del perfil de los fallecidos, tasas de mortalidad por 100 mil habitantes, años potenciales de vida perdidos y cálculo de sobremortalidad por edad en los mayores de 60 años.

**RESULTADOS:**

Se presentaron 100.758 defunciones por accidentes de tránsito en el periodo, 6.717 promedio anual, de las cuales el 18,5% ocurrió en personas de 60 años y más. Predominaron las muertes en hombres; el riesgo de morir fue de 32,1 por 100 mil personas en este rango de edad, registrando el doble del riesgo de morir que los menores 60 años.

**CONCLUSIONES:**

La población joven registra mayor proporción de defunciones, pero son los mayores de 60 años los que tienen mayor riesgo de morir. Es necesario dirigir la mirada al mejoramiento de la infraestructura y normatividad vial, a formar la población en el autocuidado y respeto de las medidas de seguridad, y a preparar a la sociedad para una población cada vez más adulta, más numerosa y más expuesta a asumir riesgos de esta naturaleza.

## INTRODUCTION

Traffic accidents cause approximately 1.24 million deaths worldwide every year. According to the World Health Organization (WHO), by 2020 this figure could be 1.9 million, increasing the gross national product in 1.0% and 3.0% to lesion treatment costs^a^ and decreasing the productivity and disability. Families generated high debts, affecting their quality of life. Although road safety is an old issue, fatalities in recent years with the most affected groups^1,2^, that is, pedestrians, cyclists and motorcyclists, are increasing, considered a global policy issue^9,b^.

Latin America has one of the highest rates of catastrophic success for pedestrians, cyclists and motorcyclists. Colombian inhabitants are about three times more likely to die in a traffic accident compared with people of Spanish countries, and four times more than English countries, since it accounts for almost 70% of deaths^c^.

The increase in this type of incident occurs because of a larger number of motor vehicles on the roads and the demographic transition. This transition led to a population structure aging^1^, in which more people are exposed to traffic accident.

The importance of traffic accidents in older adults is due to the high vulnerability associated with their age^d^. Incident or injury at advanced age^5^ can easily kill them due to their fragility to withstand a trauma and to the loss of reflexes caused by natural deterioration, different from younger populations, which have faster recovery processes. Aging can affect skills, functional capacity, memory, and learning^14^.

After homicides^2^, traffic accidents account for the most violent deaths in Colombia (a national rate of 75.3 deaths per 100,000 inhabitants in 2005). Between 2004 and 2008, the percentage of deaths due to traffic accidents in people aged 60 to 64 years was higher; however, the highest rate was the 80-year-old older adults, meaning that the risk of death increases with age^13^.

The National Institute of Forensic Medicine (INML) indicated that people over 70 years had a 46.1% mortality rate in 2011^3^. In 2013, transportation accidents totalized 48,042 cases, increasing 1.1% compared with 2012, and 13.4% in relation to 2004. The population aged 15 to 34 years had an average mortality rate of 17.1 per 100 thousand inhabitants (30.0% above the national total). The population older than 65 years (7.1%) had the worst rates, with a mean of 29.8 (125% above the national total), with 16.0% of fatalities in transport accidents^4^.

This study aimed to analyze the mortality in traffic accidents in the Colombian older adults.

## METHODS

This is a descriptive study of quantitative approach. We analyzed tendencies of the 100,758 traffic accident deaths in Colombia between 1998 and 2012, according to death certificates issued by the National Administrative Department of Statistics (DANE) registered with the codes CIE-10 (V01-V06, V09.0-V09.9, V10-V14, V19.0-19.2, V19.4-V19.6, V19.9, V20-V79, V80.3-V80.5, V81.0-V81.1, V82.0-V82.1, V83-V86, V87.0-V87.8, V88.0-V88.8, V89.0, V89.2, V89.9, Y85.0, V15-V18, V19.3, V19.8, V80.0-V80.2, V80.6-V80.9, V81.2-V81.9, V82.2-V82.9, V87.9, V88.9, V89.1, V89.3)^15^ and Population Projections of 1985-2020.

We analyzed traffic accident deaths in the total population, in those under 60 years, and in those aged 60 and older, by calculating frequency distribution, proportions, and standardized mortality rates. The direct method with the Colombian population was our standard, calculation of excess mortality in the population over 60 years, potential years of life lost (PYLL), using the ages from one to 70, as Romeder and McWhinnie suggested^12^. This allows us a broad view of the relative importance of the most relevant causes of premature mortality and risk ratio (RR), estimated as a coefficient between the years lost in each sex.

With the SPSS version 21 package, we processed the information and used the ArcGis 10 software (licenses from the Universidad CES) for georeferencing; in addition to the EPIDAT 3.1 program (free distribution).

## RESULTS

Among the 100,758 Columbian traffic accident deaths between 1998 and 2012, 18.5% were older adults. Most deaths were men (79.5%) and 74.1% were 60 years or older. The state of Antioquia had 15.1% of total deaths and Valle del Cauca had 15.2% of the older adult population deaths. The state of Vaupés showed less traffic accident deaths in the total population (eight); and Amazonas had less deaths in relation to older adults (two).

**Figure 1:**
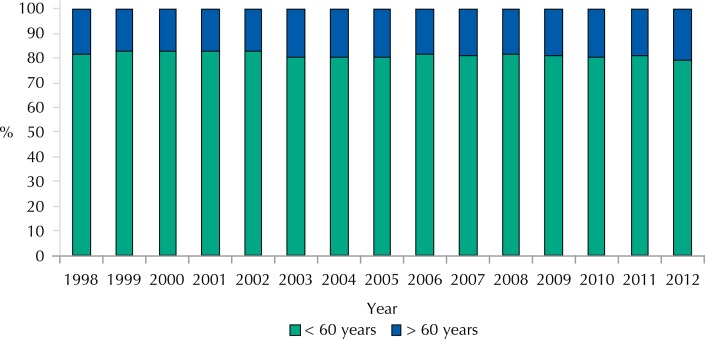
Proportion of deaths due to traffic accidents in agreement with the year in the population under and over 60 years. Colombia, 1998-2012.

Source: National Administrative Department of Statistics. Records of death and projected population. Calculations of the researchers.

The proportion of traffic accident deaths in people aged 60 years and over ranged from 17.0% to 20.6% in relation to total deaths during the study years. The year 2012 showed the highest percentage of deaths in this age group (Figure 1).

The probability of traffic accident death was 15.7/100 thousand inhabitants in the total population, presenting the highest risk in 1998 (21.1/100 thousand inhabitants). People less than 60 years old showed a risk of 14.0/100 thousand people of these ages, presenting the highest risks in 1998 and 1999, with rates of 18.8 and 17.3, respectively. In adults aged 60 years and over, the total risk was 32.1/100 thousand people. The years 1998 and 1999 had the highest rates (47.6 and 39.9, respectively) (Table 1).

**Table 1:** Risk of death (per 100,000 inhabitants) due to traffic accidents in agreement with the year and age group. Colombia, 1998-2012.

Year	Age						Total		

	Under 60 years			60 years or over					
		
	Deaths	Population	Rate	Deaths	Population	Rate	Deaths	Population	Rate
1998	6.773	36.030.897	18.8	1.502	3.153.559	47.6	8.275	39.184.456	21.1
1999	6.328	36.486.066	17.3	1.296	3.244.732	39.9	7.624	39.730.798	19.2
2000	6.041	36.957.726	16.4	1.254	3.337.837	37.6	7.295	40.295.563	18.1
2001	5.914	37.384.851	15.8	1.219	3.428.690	35.6	7.133	40.813.541	17.5
2002	5.761	37.809.423	15.2	1.210	3.519.401	34.4	6.971	41.328.824	16.9
2003	5.365	38.235.959	14.0	1.292	3.613.000	35.8	6.657	41.848.959	15.9
2004	5.196	38.658.244	13.4	1.252	3.710.245	33.7	6.448	42.368.489	15.2
2005	4.889	39.073.139	12.5	1.174	3.815.453	30.8	6.063	42.888.592	14.1
2006	5.168	39.502.071	13.1	1.166	3.903.885	29.9	6.334	43.405.956	14.6
2007	5.376	39.906.633	13.5	1.258	4.020.296	31.3	6.634	43.926.929	15.1
2008	5.313	40.294.141	13.2	1.187	4.157.006	28.6	6.500	44.451.147	14.6
2009	5.340	40.669.470	13.1	1.263	4.309.362	29.3	6.603	44.978.832	14.7
2010	4.861	41.036.904	11.9	1.177	4.472.680	26.3	6.038	45.509.584	13.3
2011	4.741	41.416.207	11.5	1.126	4.628.394	24.3	5.867	46.044.601	12.7
2012	5.012	41.788.866	12.0	1.304	4.792.957	27.2	6.316	46.581.823	13.6

Total	5.472	39.016.706	14.0	1.245	3.873.833	32.2	6.717	42.890.540	15.7

Source: National Administrative Department of Statistics. Records of death and projected population. Calculations of the researchers.

**Figure 2:**
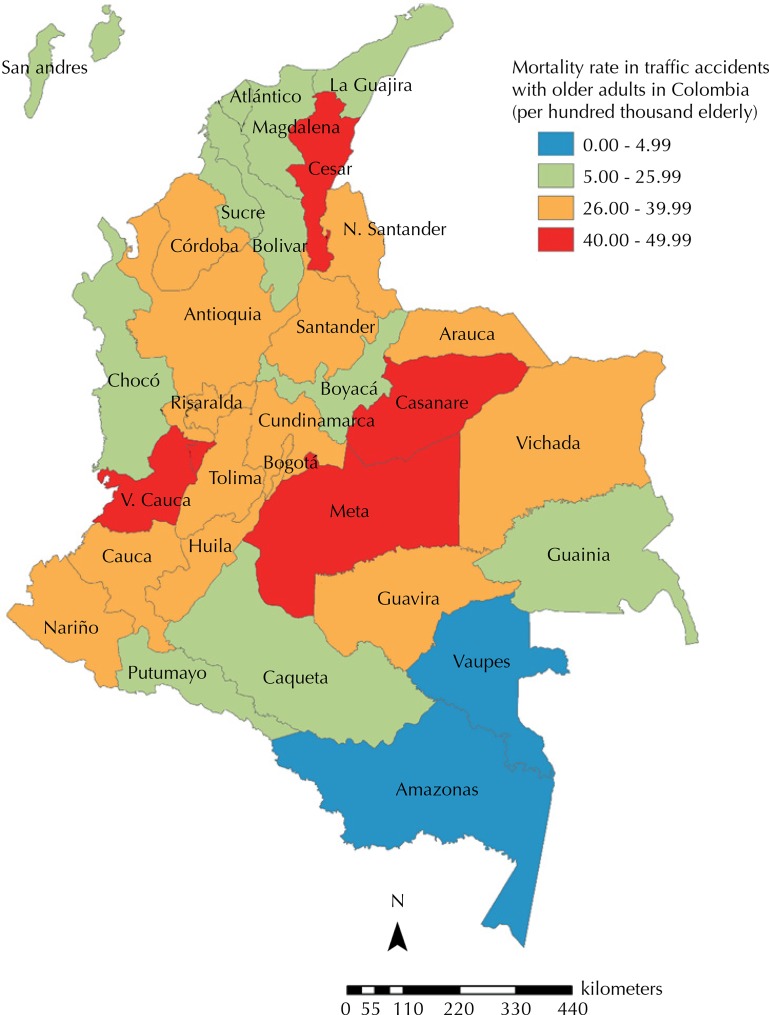
Mortality rate due to traffic accident in older adult Colombia, 1998-2012.

Source: National Administrative Department of Statistics. Records of death and projected population. Calculations of the researchers.

The states with the highest rates for older adults were Meta (49.5/100 thousand), Valle del Cauca (46.4/100 thousand) and Casanare (44.8/100 thousand). Casanare showed the highest mortality rate (25.9/100 thousand people). The lowest mortality rate due to traffic accidents occurred in the state of Vaupés (1.4/100 thousand people in the total population and 0.0/100 thousand people aged 60 years and over) (Figure 2).

**Figure 3:**
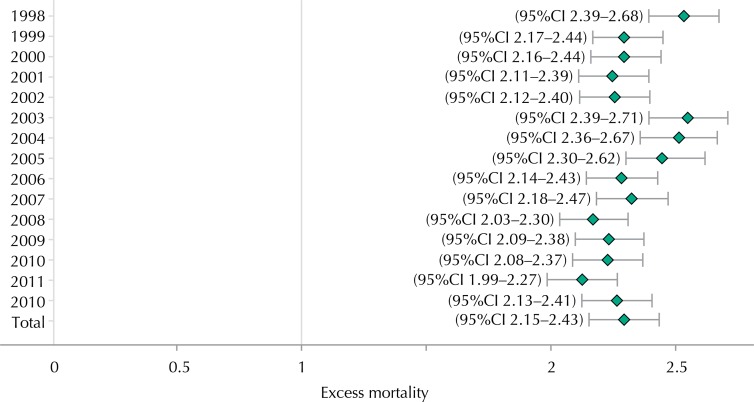
Excess mortality due to traffic accident in people aged 60 years or more in agreement with year of death. Colombia, 1998-2012.

Source: National Administrative Department of Statistics. Records of death and projected population. Calculations of the researchers.

During the 15 years of the study, people aged 60 years or older had twice the risk compared with those younger than 60 years (EM > 60 = 2.3), with the largest difference in 2003 (EM > 60 = 2.55) (Figure 3).

The highest rate of potential years of life lost (per 1,000 inhabitants) due to traffic accident occurred in 1998 (PYLL = 10.0), followed by 1999 (PYLL = 9.1) and 2000 (PYLL = 8.6). The PYLL rate decreased from 1998 to 2012; in the latter year the rate was 5.9 (Table 2).

**Table 2:** Adjusted rate of potential years of life lost (per 1,000 inhabitants) due to traffic accident, in agreement with the year of death and sex. Colombia, 1998-2012.

Year	Total	Men	Women	RR
1998	10.0	16.1	4.1	3.9
1999	9.1	14.7	3.8	3.9
2000	8.6	13.9	3.4	4.1
2001	8.3	13.2	3.4	3.8
2002	7.9	12.8	3.2	4.0
2003	7.3	11.6	3.1	3.7
2004	7.0	11.1	2.9	3.9
2005	6.4	10.3	2.6	4.0
2006	6.6	10.6	2.8	3.8
2007	6.8	11.0	2.6	4.2
2008	6.6	10.8	2.5	4.3
2009	6.5	10.6	2.5	4.2
2010	5.8	9.5	2.2	4.3
2011	5.6	9.2	2.1	4.3
2012	5.9	9.7	2.1	4.6

Source: National Administrative Department of Statistics. Records of death and projected population. Calculations of the researchers.

The rate of potential years of life lost declined and the risk ratio between men and women increased over the years. The lost years for men reduced significantly compared with the years for women, which remained more stable. Therefore, the risk ratio increased the ratio up to four times a year lost for women in 2012 (RR = 4.6) (Table 2).

## DISCUSSION

People over the age of 60 had twice the risk of death due to traffic accident than those under 60. This is similar from other studies’ results, in which people between 60 and 74 years die most from injuries caused by vehicle incidents^11^.

During the study, the state Coloque had the highest mortality rate due to traffic accident in the older adults. The total and the older adult populations showed deaths more frequent in men (74.0%) than in women. An INML 2004-2008 study found that for each older woman that dies, four men die for this reason^13^, a situation similar to the 1998-2012 study that associated 74.1% deaths to men. In Cali (1993-1997), men had up to three times the women rates; the majority of these adults were pedestrian, representing 90.0% of the deaths^11^.

In Cuba, deaths of over 60 years represent 30.4%^10^. Traffic accidents cause cranioencephalic trauma in the older adults, and men have the greatest risk of suffering from it due to their more active life and greater contact with the outside^7^.

Since the general population and the older adults are concerned with the traffic accident mortality, the mortality rates decreased during the years of analysis. In 1998, there were 21.1/100 thousand inhabitants and 47.6/100 thousand older adults, and in 2012, this rate fell to 13.6/100 thousand inhabitants and 27.2/100 thousand older adults. This is positive to the initiatives implemented in recent years to reduce deaths and disabilities occurring in traffic accidents.

A study conducted by legal medicine also showed mortality rates decrease. It observed that deaths decreased between 2000 and 2005, but not statistically significant^2^. Despite the reductions, the high fatality rate for older adults remains stable. This age group has the highest probability of death in transport accidents (29.8/100 thousand inhabitants), according to a study from 2013, being 125.0% above the national rate for that year^4^.

The decrease in mortality rates due to traffic accidents may exist due to the state interest. Measures were implemented such as: greater road signs, control of alcohol consumption, greater control in the issuance of driver’s licenses, increase in pedagogical and economic fines for users who violate traffic regulations, issuance of restrictive and preventive laws, surveillance cameras in all the large and medium-sized cities, examinations of alcoholic levels for drivers, requirement of helmets and reflective vests, among others^2,5^.

Given this situation, the risk of older adults to die in a traffic incident is constant^6^, probably because many of the measures are not applied, since they die mainly on public roads as pedestrians^8,e^.

Men are more likely to die than women^2,5,8^, due to increased exposure to motorcycle, with the majority of people under 60 dying, thereby increasing their potential years of life lost. Due to lack of handling of road safety information, the states showed differences, thus underreporting information of fatal and injured victims may be occurring^f^. The WHO states that quality data, detailed knowledge of the road, and how injuries occur, are necessary to guide road safety policies to determine interventions. This is desirable but unreal in many low-income countries where traffic data lack to reflect the realities of road accident and fatality. Research in this area helps to discover the real need for intervention^12^.

The impact of population aging also appeared in other Latin America countries; prolongation of life and improvement of quality of life should occur in all age groups. Countries should guide the policies to connect demographic trends of recent years and improve the life conditions of the older adult population. They have biological worsening in physical and psychological health, which worsen when the external situations can affect negatively their health.

Physiological processes associate with aging, such as visual acuity, hearing loss, prolonged reaction time, and impaired balance and gait. The country performed educational and infrastructure road safety policies and programs, as well as friendly roads for the older adults, which includes easily access bridge for pedestrians, timed traffic lights for pedestrians, healthy environments. All social spheres need policies to generate actions of greater impact in relation to drivers, passengers and pedestrians^f,g^.

This study could present limitations because of the information sources, mainly due to the under-registration of 21.8% deaths in Colombia in 2012, according to PAHO^h^. However, the results are valid, since it is the country’s official information.

Traffic accidents mainly affect the young population. Older adults increased proportionately, increasing the risk of dying on public roads. Colombia needs measures that aim at improving the efficiency and sustainability of road safety policies, programs and interventions, and increasing road safety for this population, along with issues such as speed control, one of the main influence factors on gravity and increase in transport accidents.

Timely assistance to people involved in traffic accidents in reasonable times can prevent a significant number of deaths, especially older adults who are more likely to die in a traffic incident, because of their fragility or because they are pedestrians.

## Footnotes

a Organización Panamericana de la Salud. Centro de prensa. Lesiones causadas por el tránsito. 2015 [cited 2015 Feb 26]. Available from: http://www.who.int/mediacentre/factsheets/fs358/es/

b Organización Mundial de la Salud. Plan mundial para el decenio de acción para la seguridad vial 2011-2020. Geneva: World Health Organization; 2011 [cited 2015 May 6]. Available from: http://www.who.int/roadsafety/decade_of_action/plan/spanish.pdf

c Ferrer A, Smith R, Espinosa O, Cuellar M, Raffo V. Análisis de la capacidad de gestión de la seguridad vial [Internet]. The World Bank Group; 2013 [cited 2015 May 5]. Available from: http://www.worldbank.org/content/dam/Worldbank/document/LAC/Analisis%20de%20la%20Capacidad%20de%20Gestion%20de%20la%20Seguridad%20Vial%20-%20Colombia%202013.pdf

d Organización Mundial de la Salud. Envejecimiento y ciclo de vida. Datos interesantes acerca del envejecimiento. Ginebra: Organización Mundial de la Salud; 2015 [cited 2015 Feb 26]. Available from: http://www.who.int/ageing/about/facts/es/

e Corporación Fondo de Prevención Vial. El legado de la seguridad vial en Colombia: informe de gestión. 2015 [cited 2015 Sep 28]. Available from: http://www.fpv.org.co/uploads/documentos/libreria/informe_gestion_fpv.pdf

f Ministerio de Transporte (CO). Resolución 1282, de 30 de marzo de 2012. Por la cual se adopta el Plan Nacional de Seguridad Vial 2011-2016. Bogotá: Ministerio de Transporte; 2012 [cited 2015 Sep 28]. Available from: http://www.alcaldiabogota.gov.co/sisjur/normas/Norma1.jsp?i=46774

g Congreso de Colombia. Ley Nº 1503 del 29 de diciembre de 2011. Por la cual se promueve la formación de hábitos, comportamientos y conductas seguros en la vía y se dictan otras disposiciones. Bogotá; 2011 [cited 2015 Sep 28]. Available from: http://www2.igac.gov.co/igac_web/normograma_files/ley%201503%20de%202011.pdf

h Organización Panamericana de la Salud. Situación de Salud en las Américas: 20 anõs: indicadores básicos 2014. Washington (DC): Organización Panamericana de la Salud; 2014 [cited 6 May 2015]. Available from: http://www.paho.org/hq/index.php?option=com_content&view=article&id=7170%3A2012-health-situation-americas-health-indicators-2014&catid=2394%3Aregional-health-observatory-reports&Itemid=2395&lang=es
